# Supplementary figures and images for: Platelet-Membrane-Encapsulated Carvedilol with Improved Targeting Ability for Relieving Myocardial Ischemia–Reperfusion Injury
Source: Membranes (Basel). 2022 Jun 10;12(6):605. doi: 10.3390/membranes12060605 (PMC9227294; doi:10.3390/membranes12060605)

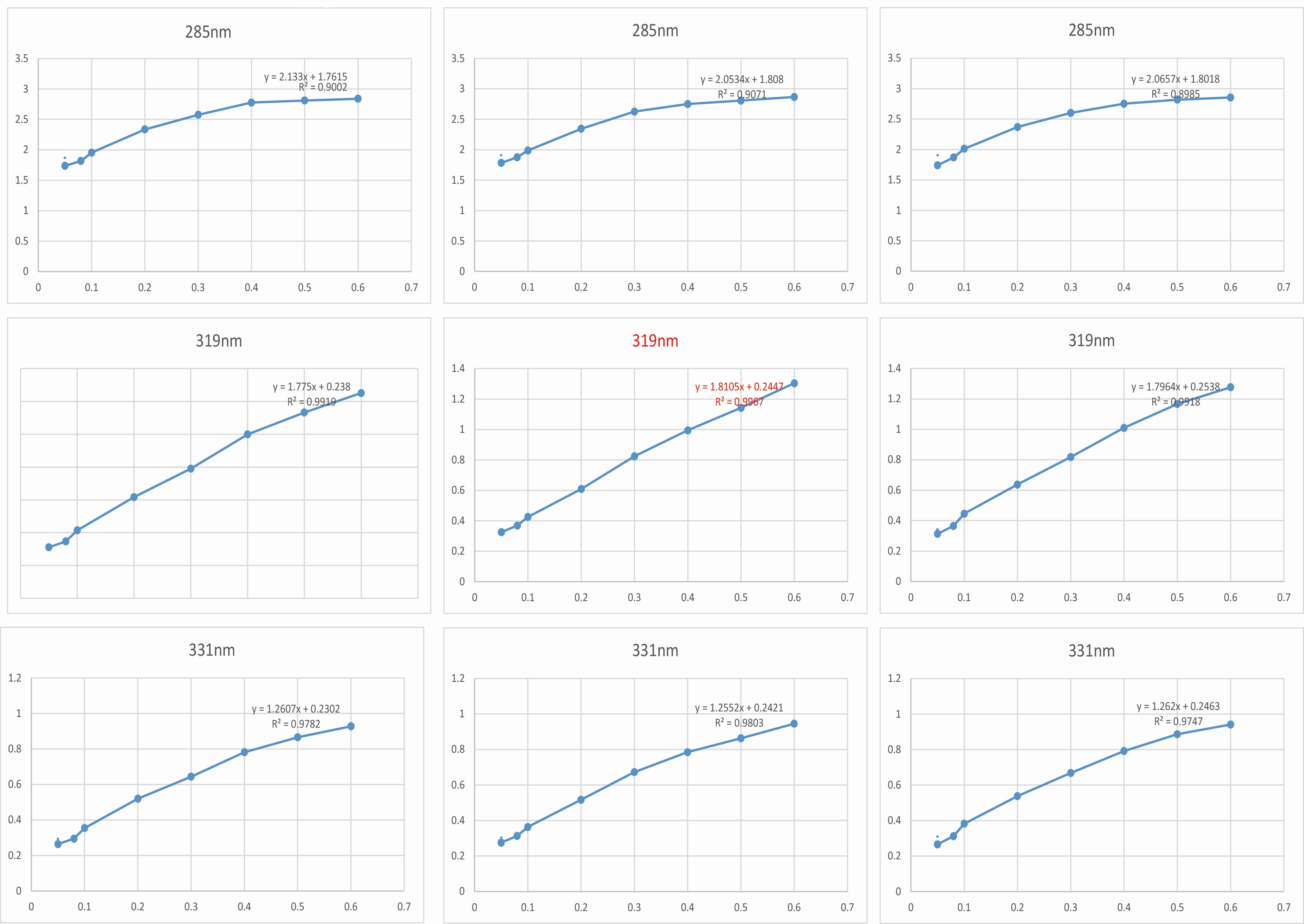

Supplement: Supplementary file 1 [file membranes-12-00605-s001.zip › membranes-1716366-supplementary/Figure S1.jpg]
